# Extracellular Vesicle-Derived miRNAs as Diagnostic Biomarkers for Pancreatic Ductal Adenocarcinoma: A Systematic Review of Methodological Rigour and Clinical Applicability

**DOI:** 10.1177/11772719251381960

**Published:** 2025-10-25

**Authors:** Ryhan Divyang Patel, Bhavik Patel, Tatjana Crnogorac-Jurcevic

**Affiliations:** 1 Imperial College London School of Medicine, UK; 2 University of Surrey, Guildford, UK; 3 Queen Mary University of London, UK

**Keywords:** pancreatic ductal adenocarcinoma, extracellular vesicles, microRNAs, diagnostic biomarkers, systematic review, QUADAS-2, MISEV 2018, liquid biopsy, early cancer detection

## Abstract

**Background:**

Pancreatic ductal adenocarcinoma (PDAC) is a highly lethal malignancy with currently limited early detection options. Extracellular vesicle (EV)-derived microRNAs (miRNAs) have gained interest as non-invasive diagnostic biomarkers due to their stability in circulation and tumour-specific profiles. However, the methodological robustness of existing literature remains unclear.

**Objectives:**

To systematically evaluate the diagnostic accuracy and methodological quality of studies investigating EV-derived miRNAs for PDAC detection, with a particular focus on adherence to established EV characterisation guidelines.

**Design:**

Systematic review registered with PROSPERO (CRD42024501503) and conducted in accordance with PRISMA 2020 reporting standards.

**Methods:**

We searched PubMed, EMBASE, Medline and Cochrane for original human studies published up to February 1, 2025, evaluating EV-derived miRNAs in biofluids from PDAC patients. Eligible studies reported diagnostic accuracy metrics (sensitivity, specificity, Area Under the Curve (AUC)). Methodological quality was assessed using the QUADAS-2 tool, and EV validation was scored against the Minimal Information for Studies of Extracellular Vesicles 2018 (MISEV) 2018 checklist (updated 2023).

**Results:**

Fifty-six studies were included. Plasma and serum were the most commonly used biofluids. The most frequently evaluated individual miRNAs were miR-21 (13 studies), miR-10b (9 studies), and miR-451a (7 studies). Although several studies reported high diagnostic performance (AUCs up to 0.99), MISEV adherence was limited: only 23.1% of miR-21 studies demonstrated strong EV validation, and >70% of all studies lacked EV quantification or protein marker analysis. Multi-miRNA panels achieved higher AUCs (often > 0.85) but typically scored poorly on EV characterisation. Only 2 of 56 studies included external validation, and 54 studies lacked blinding, contributing to substantial risk of bias.

**Conclusion:**

EV-derived miRNAs are promising PDAC biomarkers, but progress is hindered by inconsistent methods, poor EV validation, and minimal external verification. Translation to clinical use requires robust EV characterisation, standardised workflows, and prospective multi-cohort studies.

## Plain Language Summary

Pancreatic cancer is often diagnosed at a late stage and is one of the hardest cancers to treat. Researchers are exploring ways to detect it earlier, especially through simple blood tests. One promising method involves studying tiny particles in the blood called extracellular vesicles (EVs). These particles can carry small molecules known as microRNAs (miRNAs), which may signal the presence of cancer. In this study, we reviewed 56 research papers that tested EV-derived miRNAs as potential early detection tools for pancreatic cancer. Many of these studies reported promising results, including high accuracy in distinguishing cancer patients from non-cancer patients. However, most did not follow key international quality guidelines called the Minimal Information for Studies of Extracellular Vesicles (MISEV) 2018 standards. These standards help ensure that EVs are real, uncontaminated, and properly measured. We also found that many studies did not use “blinding” which is a process where researchers analysing the samples do not know which ones are from cancer patients. This helps prevent unconscious bias, where expectations might unintentionally affect how results are interpreted. Additionally, very few studies tested their findings in new groups of patients, which is important for confirming that the results are reliable. Our review shows that while EV-miRNAs could be useful for detecting pancreatic cancer earlier, the overall quality of current research is limited. Future studies need to follow international standards more closely, use blinding, and include independent testing before these methods can be confidently used in clinical practice.

## Introduction

Pancreatic ductal adenocarcinoma (PDAC) is the most common malignancy of the pancreas, and one of the deadliest cancers.^1^ In the UK around 10 500 patients are diagnosed with the disease every year.^2^ Due to steadily increasing incidence and by association mortality, PDAC poses an unmet clinical challenge causing a significant burden on both patients and healthcare providers.^3,4^ This foreboding outlook is underpinned by nonspecific symptoms, late-stage diagnosis and limited therapeutic options. Around two-thirds of patients have metastatic disease at diagnosis, which is associated with an exceedingly poor 3-year survival of <5%.^5^ Research focussing on aetiology and early detection thus offers best hope for changing the course of this disease. Currently, there are no clinically validated biomarkers for early detection of PDAC - Carbohydrate antigen 19-9 (CA19-9) is used in the management of patients with PDAC but offers an inadequate sensitivity (SN), specificity (SP) and positive predictive value (PPV) to warrant its use as an early biomarker.^6^

Extracellular vesicles (EVs) are lipid bilayer membrane-enclosed vesicles that are secreted from cells into the extracellular space and can be isolated from plasma, bile, urine and other biofluids.^7^ The interest in EVs is rapidly increasing due to improving isolation techniques and their overexpression in cancer.^8^ PDAC-derived EVs have already been shown to initiate pre-metastatic niche formation in the liver, a key step in the development of metastatic disease.^9^ The EV load can include various bioactive molecules such as lipids, proteins and microRNAs (miRNAs).^10^

MicroRNAs (miRNAs) are small (≈22 nucleotide) non-coding RNAs that regulate gene expression, usually through translational repression or by reducing messenger RNA stability.^11^ They can act as tumour suppressors or oncogenes and miRNA profiling can be used to distinguish different cancer subtypes.^12^ Furthermore, miRNAs have also been shown to function as ligands which directly bind Toll-like receptors and activate pro-metastatic signalling pathways.^13^ MiRNAs are particularly interesting biomarkers due to their presence and stability in the circulation. This is especially the case when they are contained within EVs, which can further shield them from enzymatic degradation.^14^

This review focuses on EV miRNAs as biomarkers in PDAC, their isolation methodology, downstream target genes and diagnostic accuracy. An additional aim was to gain oversight as to whether current literature is meeting the standards required for consistency and reproducibility as defined in the 2018 guidelines by the International Society for Extracellular Vesicles.^15^

## Methods

### Search Strategy

This systematic review is registered in PROSPERO (CRD42024501503) and adhered to the Preferred Reporting Items for Systematic Reviews and Meta-Analyses (PRISMA) 2020 guidelines.^16^ It was not feasible to conduct a meta-analysis due to substantial heterogeneity in study methodologies, biofluid sources and reporting of diagnostic performance metrics. The authors conducted comprehensive and systematic search on 4 separate electronic databases: PubMed, Medline, EMBASE, and Cochrane, for papers published between 1946 and February 1, 2025, when the searches were performed. The search terms for each database are provided in Supplemental Information.

Manual retrieval of the references from included articles was also performed to identify additional relevant studies.

The inclusion and exclusion criteria were pre-specified to determine the eligibility of studies for the review. Studies were included if they: (1) investigated PDAC; (2) utilised non-invasive sample types such as plasma, serum, urine, saliva or stool; (3) were original research articles; and (4) involved human participants. Studies were excluded if they: (1) did not focus on PDAC; (2) did not specify the pancreatic cancer subtype; (3) relied on invasive sampling methods (eg, tissue biopsy); (4) did not report SN, SP and AUC; (5) were abstracts, conference proceedings, NHS reports or review articles; (6) were non-English publications; or (7) involved non-human data.

All retrieved articles were imported into Rayyan software for systematic review (https://www.rayyan.ai/ accessed on 1st February 2025). Duplicates were removed, and relevant articles were screened based on title and abstract.

### Data Extraction and Quality Assessment

Data extraction was as follows: year of publication, first author, country of study, total number of samples, PDAC stage (if mentioned), number and health status of controls (if stated), biofluid type, method of EV extraction, miRNA concentration measuring method, miRNA biomarker investigated, diagnostic accuracy parameters, proposed biomarker role (diagnostic or prognostic). Discrepancies between the reviewers were resolved through consensus.

The adherence of papers’ methodologies to the Minimal Information for Studies of Extracellular Vesicles (MISEV) 2018 criteria along with updated sections found in 2023 criteria^15^ were tested in order to assess methodological rigour. The criteria used to determine numerical scores is provided in Box 1.

**Box 1.** ISEV 2018 Modified EV Characterisation Checklist Score Including Updated Sections Aligning with 2023 Version.

**Quantification (3 points)** (performed via Nanoparticle Tracking Analysis (NTA)/Bicinchoninic Acid Assay(BCA)/Tunable Resistive Pulse Sensing (TRPS)/Microfluidic Resistive Pulse Sensing (MRPS). 1 point is given for each data category below: • Source: total starting volume of biofluid • EV preparation: global quantification of EVs should be provided. The most commonly used are total protein amount and total particle number. • Ratio of the two quantification figures (e.g. protein to particle ratio) **Global characterisation (3 points)** **(**performed via Western blotting or similar method) 1 point is given for various types of protein: • Transmembrane or GPI-anchored protein localised in cells at plasma membrane or endosomes: Non-tissue specific or cell/tissue specific. • Cytosolic protein with membrane-binding or - association capacity: With lipid or membrane protein-binding ability or promiscuous incorporation in EVs (and possibly exomeres). • Assessment of presence/absence of expected contaminants Lipoproteins (produced by liver, abundant in plasma, serum) or protein and protein/nucleic acid aggregates. **Single EV characterisation (2 points)** 1 point is given for each technique below: • Images of single EVs by wide-field and close-up: e.g. electron microscopy, scanning probe microscopy, super-resolution fluorescence microscopy • Non-image-based method analysing large numbers of single EVs: NTA, TRPS, FCS, high-resolution flow cytometry, multi-angle light-scattering, Raman spectroscopy, etc.

The analysis of the findings from the included manuscripts was conducted, focussing on grouping studies assessing the most commonly reported miRNAs and examining similarities and differences in adherence to MISEV criteria. Throughout the analysis, scores were categorised as low (0-2), moderate (3-4) or strong adherence (5-8). These thresholds reflect the extent to which studies incorporated essential EV characterisation steps, with scores of 5 or above indicating adherence to at least 2 of the 3 major validation criteria (quantification, global characterisation and single EV characterisation). This allowed for thorough evaluation of the robustness of current data exploring diagnostic potential of various miRNA biomarkers in PDAC.

Risk of bias was also conducted following the Quality Assessment of Diagnostic Accuracy Studies (QUADAS-2) tool for evaluating diagnostic studies.^17^ The domains of patient selection, index testing and reference standard reporting were assessed, however the flow and timing domain was excluded due to lack of relevance to these studies.

## Results

**Figure 1.**
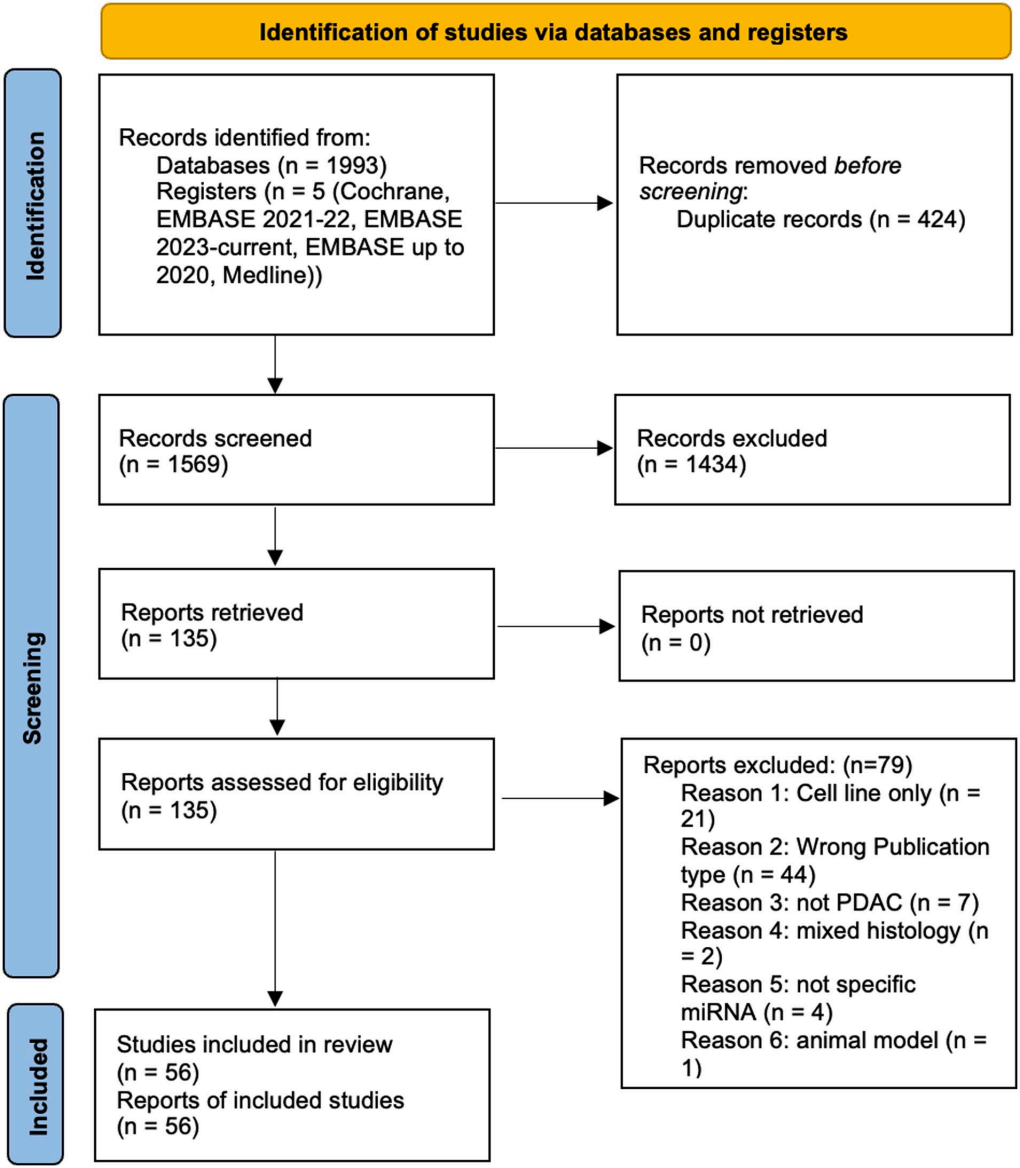
PRISMA flow diagram of the search strategy and study selection.

A PRISMA flow diagram demonstrating the search strategy and study selection criteria that were applied according to^18^ is shown in Figure 1.

The key methodological characteristics of the incorporated studies, including biofluid type, EV isolation and miRNA quantification methods, control group composition and PDAC staging are summarised in Table 1. It is evident that plasma and serum were the most commonly analysed biofluids, with a smaller number of studies utilising pancreatic juice or urine. A range of EV isolation methods was employed, including ultracentrifugation, polymer-based precipitation kits and size-exclusion chromatography. miRNA extraction was performed using various commercial RNA isolation kits, and RT-qPCR was the most frequently used platform for miRNA quantification. Fewer studies employed microarray or next-generation sequencing approaches. Reporting of input biofluid volumes, elution conditions, and RNA quality control measures varied considerably. Control groups consisted of healthy individuals, patients with benign pancreatic conditions, or mixed non-cancer cohorts.

**Table 1.** Study Characteristics of Selected 56 Manuscripts.

miRNA biomarker	Biofluid	Total number of participants	Method of EV extraction	miRNA concentration measuring method	Sensitivity (%)	Specificity (%)	AUC	Proposed biomarker role	Total MISEV score breakdown	Reference
miR-21, miR-196a, miR-451a and miR-1246	Blood	50 (35 PDAC, 15 H)	EV centrifugation	Endonucleolytically Exponentiated Rolling Circle Amplification with CRISPR–Cas12a’ (EXTRA-CRISPR)	NS	NS	0.853	Diagnostic	5 (1 + 2 + 2)	Yan et al.^19^
miR-21					NS	NS	0.767			
miR-196a					NS	NS	0.693			
miR-451a					NS	NS	0.793			
miR-1246					NS	NS	0.677			
miR-210	Blood	73 PDAC	Precipitation: total exosome isolation kit (Thermo, USA) according to the manufacturer’s protocol	qRT-PCR	NS	NS	0.896	Prognostic	2 (0 + 1 + 1)	Wu et al.^20^
miR-95-3p and miR-26b-5p	Blood	90 PDAC	Precipitation: 3D Medicines isolation reagent	qRT-PCR	84.1	96.6	NS	Diagnostic	5 (0 + 3 + 2)	Guo et al.^21^
miR-95-3p					NS	NS	Training cohort = 0.946. Test cohort = 0.875.			
miR-26b-5p					NS	NS	0.908			
miR-155-5p	Blood	10 (5 PDAC, 5 H)	Ultracentrifugation	RT-qPCR	NS	NS	NS	Diagnostic	0 (0 + 0 + 0)	Girolimetti et al.^22^
miR-27a-3p						NS	NS			
let-7a-5p						NS	NS			
miR-221-3p						NS	NS			
miR-23b-3p						NS	NS			
miR-193a-3p						NS	NS			
miR-196a, miR-196b and miR-1246	Plasma	30 (15 PDAC, 15 H)	Precipitation: ExoQuick (System Biosciences)	RT-qPCR	NS	NS	NS	Prognostic	3 (0 + 1 + 2)	Xu et al.^23^
miR-196a					NS	NS	0.81			
miR-196b					NS	NS	0.71			
miR-1246					NS	NS	0.73			
miR-103b, miR-23a-3p, miR-409-3p, miR-224-5p, miR-1299	Plasma	204 (89 PDAC, 71H, 44 IPMN/CP)	Affinity-based isolation: EV’s magnetically labelled via biotinylated antibodies and anti-biotin ultrapure 50-nm-diameter nanoparticles (Miltenyi Biotec). Pipetted into the human plasma samples, incubated and loaded onto the reservoir of the TENPO device and pushed through it via Braintree Scientific syringe pump	qPCR	88	95	0.95	Diagnostic	1 (1 + 0 + 0)	Yang et al.^24^
miR-103b					NS	NS	NS			
miR-23a-3p					NS	NS	NS			
miR-409-3p					NS	NS	NS			
miR-1299					NS	NS	NS			
miR-224-5p					NS	NS	NS			
miR-30b-5p	Plasma	48 (24 PDAC, 24 H)	Centrifugation	RT-qPCR	NS	NS	NS	Diagnostic	2 (0 + 1 + 1)	Chen et al.^25^
Exosomal miR-30b-5p					NS	NS	0.934			
Total miR-30b-5p					NS	NS	0.9826			
miR-130b-5p, miR-133a-3p, miR-195-5p, miR-432-5p, miR-1229-3p, miR-1273f	Plasma	210 PDAC	Filtration: exosomal RNA was isolated from 400 μL plasma, using an exoRNeasy Midi Kit (Qiagen, Valencia, CA) according to the manufacturer’s instructions	qRT-PCR	72	85	NS	Prognostic	1 (1 + 0 + 0)	Nakamura et al.^26^
miR-130b-5p					NS	NS	0.81			
miR-133a-3p					NS	NS	NS			
miR-195-5p					NS	NS	NS			
miR-1229-3p					NS	NS	NS			
miR-432-5p					NS	NS	NS			
miR-1273f					NS	NS	NS			
exmiR-21, exmiR-10b and exmiR-212-3p	Plasma	101 (36 PDAC, 65 H)	Precipitation: Invitrogen™ Total Exosome Isolation Kit	TCLN biochip	NS	NS	NS	Diagnostic and Prognostic	3 (0 + 2 + 1)	Pu et al.^27^
ex-miR-21					NS	NS	NS			
ex-miR-10b					NS	NS	0.7171			
ex-miR-212-3p					NS	NS	0.6543			
ex-miR-21, ex-miR-10b					NS	NS	NS			
miR-10b	Plasma	15 (5 PDAC, 5 H, 5 CP)	Precipitation: total exosome isolation reagent (Life Technologies, USA)	SERS biosensor	NS	NS	0.791	Diagnostic	4 (0 + 2 + 2)	Pang et al.^28^
miR-196a and miR-1246	Plasma	14 (7 PDAC, 7 H)	Precipitation: Exoquick reagent + a combination of centrifugation, ultracentrifugation	qRT-PCR	NS	NS	0.89	Diagnostic	5 (0 + 3 + 2)	Xu et al.^29^
miR-196a										
miR-1246					NS	NS	NS			
miR-21, miR-155, miR-429, miR-1290, miR-10b	Plasma	40 (20 PDAC, 20 CC)	Precipitation: Total Exosomes Isolation kit (Invitrogen, Pleasanton, CA, USA). PDAC EV’s were isolated using an affinity-based method (other)	RT-qPCR	90.48	90	NS	Diagnostic	3 (0 + 1 + 2)	Kim et al.^30^
miR-21					NS	NS	0.974			
miR-155					NS	NS	0.771			
miR-429					NS	NS	0.843			
miR-1290					NS	NS	0.867			
miR-10b					NS	NS	0.786			
miR-451a	Plasma	76 (56 PDAC, 20 H)	Ultracentrifugation method (15 000*g* for 70 min)	qRT-PCR	69.2	70.8	0.693	Diagnostic and prognostic	1 (0 + 0 + 1)	Takahasi et al.^31^
miR-122-5p, miR-125b-5p, miR-192-5p, miR-193b-3p, miR-221-3p and miR-27b-3p	Plasma	436 (216 PDAC, 220 H)	Precipitation: ExoQuick Solution	qRT-PCR	88.7	89.1	0.789	Diagnostic + prognostic	1 (1 + 0 + 0)	Zhou et al.^32^
miR-122-5p					NS	NS	0.937			
miR-125b-5p					NS	NS	0.81			
miR-192-5p					NS	NS	0.646			
miR-193b-3p					NS	NS	0.693			
miR-221-3p					NS	NS	0.775			
miR-27b-3p					NS	NS	0.625			
Exo-miR-19b	Plasma	168 (62 PDAC, 53 H, 23 CP, 30 OPT)	Density gradient: exoRNeasy Serum/Plasma Midi Kit according to the manufacturer’s instructions	qRT-PCR	PDAC patients from healthy volunteers = 85.48 PDAC from CP sensitivity = 80.65 Differentiating PDAC from OPT sensitivity = 93.55	PDAC patients from healthy volunteers specificity = 90.57 PDAC from CP specificity = 86.96 PDAC from OPT specificity = 63.33	0.656	Diagnostic	1 (1 + 0 + 0)	Wang et al.^33^
377 miRNAs were used such as EV miR-21 and miR-195, miR-484	Plasma	NS	Antibody-based + ultracentrifugation (to extract EV’s from PDAC cell culture)	Fast real-time PCR	NS	NS	Differentiating Pca patients from healthy volunteers (AUC = 0.942). Differentiating Pca from CP (AUC = 0.898). Differentiating Pca from OPT (AUC = 0.810)	Diagnostic	3 (0 + 1 + 2)	Zeöld et al.^34^
miR-10b, miR-21, miR-30c, miR-106b, miR-20a, miR-181a, miR-483, miR-let7a, miR-122	Plasma	46 (29 PDAC, 6 H, 11 CP)	Centrifugation	RT-qPCR	NS	NS	0.69	Diagnostic	3 (1 + 1 + 1)	Lai et al.^35^
miR-10b					100	100	NS			
miR-21					100	100	1			
miR-30c					100	100	1			
miR-106b					62	100	1			
miR-20a					83	100	0.85			
miR-181a					100	100	0.95			
miR-483					31	100	1			
miR-let7a					100	100	0.57			
miR-122					93	100	1			
miR-10b	Plasma	9 (3 PDAC, 3 H, 3 CP)	Ultracentrifugation	LSPR-based quantification	NS	NS	0.996	Diagnostic	4 (1 + 1 + 2)	Joshi et al.^36^
miRNA-10b	Plasma	40 (10 PDAC, 10 H, 10 HCC, 10 LC)	Ultracentrifugation method	FET sensing microsystem	90	100	NS	Diagnostic	5 (1 + 2 + 2)	Yu et al.^37^
analysed 44 miRNAS	Plasma	21 PDAC (12 metastatic disease, nine locally advanced disease)	Chromatography: SEC columns of polysaccharide resin (qEV 70 columns; IZON, Christchurch, New Zealand) following the company’s protocol	RT-PCR	NS	NS	0.98	Diagnostic	4 (2 + 1 + 1)	Vannini et al.^38^
miR-664a-3p, miR-652-5p, miR-33a-3p, miR-5010-3p, miR-335-3p, miR-548e-5p, miR-940, miR-616-5p, miR-490-3p, miR-5100-3p, and miR-548d-3p	Plasma	106 (58 PDAC, 20 H, 12 CP, 12 BPT)	Precipitation: by affinity-based binding to spin columns using an exoRNeasy Serum/Plasma Kit (Qiagen, Hilden, Germany) following the manufacturer’s instructions	RT-qPCR	71.8	97	NS	Diagnostic	3 (1 + 1 + 1)	Pu et al.^39^
miR-21	Plasma	29 (16 PDAC, 13 H)	Precipitation: Vn96 peptide (ME kit [plasma], Biosynth, US)	droplet digital PCR (ddPCR)	NS	NS	0.77	Analysis of copy number versus control rather than AUC for diagnosis	1 (1 + 0 + 0)	Roy et al.^40^
miRNA-150					NS	NS	NA			
miR-374b					NS	NS	NA			
miR-744					NS	NS	NA			
miR-10b miR-21 miR-16 miR-155 miR-196a miR-1246	Plasma	36 (2-PDAC, 6 CP, 10 H)	Other: ETFB -based encoded fusion strategy	qRT-PCR	NS	NS	0.98	Diagnostic	3 (1 + 0 + 2)	Feng et al.^41^
miR-10b					NS	NS	0.91			
miR-21					NS	NS	0.93			
miR-16					NS	NS	0.9			
miR-155					NS	NS	0.73			
miR-196a					NS	NS	0.795			
miR-1246					NS	NS	0.895			
miR-502 miR-18a miR-106a miR-660 miR-21	Plasma	Initial screening (n = 24, 12 PDAC, 12 H) miR-1246 (n = 20, 10 PDAC, 10 H) miR-106a (n = 19 PDAC, 19 H) miR-18a (n = 40 20 PDAC, 20 H)	Other: Immunoaffinity isolation	qRT-PCR	NS	NS	NS	Diagnostic	2 (1 + 1 + 0)	Xu et al.^42^
miR-502					NS	NS	0.023			
miR-18a					NS	NS	0.011			
miR-106a					NS	NS	0.047			
miR-660					NS	NS	0.011			
miR-21					NS	NS	0.733			
miR-93-5p miR-339-3p miR-425-5p miR-425-3p	Plasma	34 (15 PDAC, 19 H)	Precipitation: PEG-based Total Exosome Isolation Kit	RT-qPCR	80	94.7	0.887	Diagnostic	0 (0 + 0 + 0)	Makler et al.^43^
miR-130a-3p miR-21-5p miR-223-3p miR-7975 miR-8069 miR-1228	Plasma	262 PDAC	Ultracentrifugation + exoRNeasy Serum/Plasma Maxi Kit (Qiagen)	RT-qPCR	NS	NS	NS	Diagnostic	1 (1 + 0 + 0)	Yang et al.^44^
miR-130a-3p					70.49	89.71	0.8674			
miR-21-5p					88.52	75	0.8997			
miR-223-3p					73.77	70.59	0.7842			
miR-7975					59.02	92.65	0.7963			
miR-8069					72.13	76.47	0.7642			
miR-1228					86.89	97.06	0.9652			
13 miRNA panel, 5 circulating miRNAs, 8 Ex-miRNAs	Plasma	292 (168 PDAC, 124 H)	Filtration: A exoRNeasy Serum/Plasma Midi Kit + miRNAeasy kit (Quiagen)	RT-qPCR	N/A	N/A	N/A	Diagnostic (early-detection)	0 (0 + 0 + 0)	Nakamura et al.^45^
All candidate Training Cohort Ex-miRNA signature					87	94	0.97			
All candidate Validation Cohort Ex-miRNA panel					79	94	0.89			
Reduced candidate Training Cohort Ex-miRNA panel					89	91	0.96			
Reduced candidate Validation Cohort Ex-miRNA panel					81	94	0.79			
miR-27b-3p miR-125b-3p miR-122-5p miR-21-5p miR-211-5p miR-19b-3p miR-205-5p	Plasma	143 (65 PDAC, 78 H)	Filtration: miRCURY^®^ Exosome Serum/Plasma Kit (Qiagen, Hilden, Germany)	RT-qPCR	NS	NS	NS	Diagnostic & Prognostic	2 (1 + 0 + 1)	Marin et al.^46^
miR-125b-3p					NS	NS	0.782			
miR-122-5p					NS	NS	0.814			
miR-205-5p					NS	NS	0.857			
miR-10b	Plasma	20\910 PDAC, 10 H)	Filtration: A exoRNeasy Serum/Plasma Midi Kit	PNA-functionalised nanochannel biosensor	NS	100	0.99	Diagnostic	3 (0 + 1 + 2)	Xiao et al.^47^
miR-106-5p miR-7975 miR-4454 miR-16-5p miR-25-3p miR-320e miR-451a	Plasma	140 (58 PDAC, 42 PanNET, 20 IPMN, 20 AVC)	Precipitation: ExoQuick system (System biosciences)	qRT-PCR	NS	NS	NS	Diagnostic	5 (2 + 2 + 1)	Vicentini et al.^48^
miR-4525 miR-451a miR-21	Plasma	75 (55 PDAC, 20 H)	Filtration: miRNeasy serum/plasma kit (Qiagen, Venlo, the Netherlands)	qRT-PCR	NS	NS	NS	Not diagnostic, looks at DFS and OS. Prognostic	2 (1 + 0 + 1)	Kawamura et al.^49^
miR-4525					81.8	86.4	NS			
miR-451a					72.7	77.3	NS			
miR-21					72.7	72.7	NS			
miR-141-3p	Plasma	61 (30 PDAC, 31 BPT)	SEC (qEVoriginal 70 nm)	Small RNA-seq + RT-qPCR (Qiagen miRCURY LNA RT Kit)	NS	NS	0.765	Diagnostic	5 (1 + 2 + 2)	Liu et al.^50^
miR-200a-3p					NS	NS	0.783			
miR-200b-3p					NS	NS	0.702			
miR-200c-3p					NS	NS	0.728			
miR-429					NS	NS	0.668			
Combined panel (miR-141-3p + miR-200a-3p + miR-200b-3p + miR-200c-3p + miR-429)					72	88	0.823 (technical)/0.970 (clinical validation)			
miR-451a	Serum	376 (191 PDAC, 90 H, 95 BPD)	Filtration: exoRNeasy Maxi Kit (Qiagen Gmbh, Hilden, Germany)	qRT-PCR	80.1	86.67	0.934	Diagnostic	3 (0 + 1 + 2)	Chen et al.^51^
let-7b-5p, miR-192-5p, miR-19a-3p, miR-19b-3p, miR-223-3p, and miR-25-3p	Serum	296 (159 PDAC, 137 H)	Precipitation: ExoQuick Exosome Precipitation Solution (System Biosciences, Mountain View, CA)	qRT-PCR	95.3	76.7	NS	Diagnostic and prognostic	1 (1 + 0 + 0)	Zou et al.^52^
let-7b-5p					79.8	59.8	0.91			
miR-192-5p					77.5	57	0.703			
miR-19a-3p					71.3	78.5	0.684			
miR-19b-3p					65.1	81.3	0.615			
miR-223-3p					78.3	91.6	0.771			
miR-25-3p					66.7	80.4	0.788			
ex-miR-21, ex-miR-191, ex-miR-451a	Serum	83 (32 PDAC, 22 H, 29 IPMN)	Precipitation: ExoQuick Solution	qRT-PCR.	NS	NS	NS	Diagnostic + Progression markers	1 (1 + 0 + 0)	Goto et al.^53^
ex-miR-21					80.8	81	NS			
ex-miR-191					71.9	84.2	0.826			
ex-miR-451a					65.6	85.7	0.788			
Serum miR-483-3p, Exosomal miR-483-3p	Serum	127 (107 PDAC, 20 H)	Precipitation: using ExoQuick Exosome Precipitation Solution	qRT-PCR	NS	NS	NS	Diagnostic + prognostic	1 (0 + 0 + 1)	Shao et al.^54^
Serum miR-483-3p					74.6	77.3	0.84			
Exosomal miR-483-3p					NS	NS	0.81			
miRNA-10b	Serum	30 (15 PDAC, 15 H)	Other: Fe3O4@TiO2 core–shell nanoparticles for exosome isolation using an external magnet.	SERS biosensor is compared to qRT-PCR	NS	NS	0.99	Diagnostic	2 (0 + 0 + 2)	Jiang et al.^55^
miR-1290	Serum	49 (28 PDAC, 21 H)	Other: CLHN-CCC	CLHN-CCC (mainly the CCC portion of the method)	92.9	85.7	0.882	Diagnostic + prognostic	5 (1 + 2 + 2)	Zhang et al.^56^
miR-451 and miR-720	Serum	99 (72 PDAC, 20 H, seven with precursor lesions	Centrifugation	RT-qPCR	NS	NS	0.94	Diagnostic	5 (1 + 2 + 2)	Verel-Yilmaz et al.^57^
miR-451					NS	NS	NS			
miR-720					NS	NS	0.9329			
miRNA-1226-3p	Serum	47 (27 PDAC, three H, 17 BPD)	Filtration: exoEasy Maxi Kit (QIAGEN)	qRT-PCR	NS	NS	1	Diagnostic	2 (0 + 1 + 1)	Wang et al.^58^
miR-21	Serum	40 PDAC	Precipitation: ExoQuick (System Biosciences)	qRT-PCR	80	90	0.74	Diagnostic	7 (2 + 3 + 2)	Wu et al.^59^
miRNA-210				qRT-PCR	83	90	0.869			
miR-21 and miRNA-210				qRT-PCR	93	80	0.823			
miRNA-192-5p	Serum	74 (44 PDAC, 12 H, seven IPMN, 11 CP)	Ultracentrifugation	qRT-PCR	NS	NS	NS	Diagnostic	3 (0 + 2 + 1)	Flammang et al.^60^
miRNA-200b	Serum	89 (56 PDAC, 22 H, 11 CP)	Differential centrifugation & Antibody based: EpCAM-specific magnetic Dynabeads	qRT-PCR	NS	NS	0.732	Diagnostic & Prognostic	2 (0 + 2 + 0)	Reese et al.^61^
13 miRNA panel, 5 circulating miRNAs, 8 Ex-miRNAs	Serum	292 (168 PDAC, 124 H)	Filtration: A exoRNeasy Serum/Plasma Midi Kit + miRNAeasy kit (Quiagen)	RT-qPCR	N/A	N/A	N/A	Diagnostic (early-detection)	0 (0 + 0 + 0)	Nakamura et al.^45^
All candidate Training Cohort Ex-miRNA signature					87	94	0.97			
All candidate Validation Cohort Ex-miRNA panel					79	94	0.89			
Reduced candidate Training Cohort Ex-miRNA panel					89	91	0.96			
Reduced candidate Validation Cohort Ex-miRNA panel					81	94	0.79			
miR-21 miR-25 miR-210 miR-16	Serum	172 (54 PDAC, 118 NMC)	QIAzol Lysis Reagent (Qiagen, Hilden, Germany, #79306) and miRNeasy Mini kit (Qiagen, Hilden, Germany, #217004)	RT-qPCR	84.2	81.5	0.91	Diagnostic	3 (1 + 1 + 1)	Nesteruk et al.^62^
miR-21					NS	NS	NS			
miR-25					NS	NS	NS			
miR-210					NS	NS	NS			
miR-16					NS	NS	NS			
miR-155					NS	NS	NS			
miR-1246 miR-4644 miR-3976 miR-4306	Serum	210 (131 PDAC, 30 H, 25 CP, 22 BPT, 12 non-PDAC)	Filtration: (miRNeasyMinikit, Qiagen, Hildesheim, Germany)	qRT-PCR	81	94	NS	Diagnostic	1 (0 + 1 + 0)	Madhavan et al.^63^
miR-17-5p miR-21 miR-155 miR-196a	Serum	98 (49 PDAC, 8 H, 22 PC, 6 BPT, 7 AC, 6 CP)	Ultracentrifguation + Precipitation: mirVana PARIS RNA isolation kit (Ambion, Austin, TX, USA)	RT-PCR	NS	NS	0.958	NS	0 (0 + 0 + 0)	Que et al.^64^
miR-17-5p					72.7	92.6	NS			
miR-21					95.5	81.5	0.887			
miR-155					NS	NS	0.897			
miR-196a					NS	NS	NS			
miR-196a	Serum	24 (12 PDAC, 12 H)	Ultracentrifugation	Solution-Gated Graphene Transistor (SGGT) sensor	NS	NS	0.98	Diagnostic	3 (1 + 0 + 2)	Song et al.^65^
miR-7977	Serum	239 (127 PDAC, 112 H)	exoEasy Maxi Kit (Qiagen) – Membrane Affinity Column	Microarray (Agilent) & RT-qPCR (GeneCopoeia)	75.47 (training)	88.89 (training)	0.825 (training)	Diagnostic	5 (1 + 2 + 2)	Chen et al.^66^
					56.06 (validation)	96.61 (validation)	0.796 (validation)			
miR-451a					75.47 (training)	77.78 (training)	0.804 (training)			
					81.82 (validation)	83.05 (validation)	0.830 (validation)			
Combined Panel (miR-7977 + miR-451a)					NS	NS	0.901 (training)			
					NS	NS	0.918 (validation)			
miR-142-3p	Serum	22 PDAC	Ultracentrifugation (UC), Size Exclusion Chromatography (SEC), Exosupur Column, Tissue Dissociation Kit (Miltenyi Biotec)	Small RNA-seq (Ion Total RNA-Seq Kit V2), RT-qPCR (Thermo Fisher)	NS	NS	NS	Diagnostic and Prognostic	5 (1 + 2 + 2)	Zhu et al.^67^
miR-148a-3p					NS	NS	NS			
Combined Panel (miR-142-3p + miR-148a-3p + CA19-9)					NS	NS	0.747			
miR-21	Serum	60 (30 PDAC, 30 H)	Ultracentrifugation	RT-qPCR	85	90	0.92	Diagnostic	5 (1 + 2 + 2)	He et al.^68^
ex-miR-21 and ex-miR-155	Pancreatic Juice	35 (27 PDAC, 8 CP)	Ultracentrifugation	qRT-PCR	96	75	0.901	Diagnostic	5 (1 + 2 + 2)	Nakamura et al.^69^
ex-miR-21					81	88	NS			
ex-miR-155					89	88	0.9			
miR-21, miR-155, miR-429, miR-1290, miR-10b	Pancreatic Juice	40 (20 PDAC, 20 CC)	Precipitation: Total Exosomes Isolation kit (Invitrogen, Pleasanton, CA, USA). PDAC EV’s were isolated using an affinity-based method (other)	RT-qPCR	90.48	90	NS	Diagnostic	3 (0 + 1 + 2)	Kim et al.^30^
miR-21					NS	NS	0.974			
miR-155					NS	NS	0.771			
miR-429					NS	NS	0.843			
miR-1290					NS	NS	0.867			
miR-10b					NS	NS	0.786			
miR-301a-3p	Pancreatic Juice	62 (50 PDAC)	Precipitation: Exoquick reagent	qRT-PCR.	NS	NS	NS	Prognostic	4 (0 + 2 + 2)	Wang et al.^70^
miR-21 miR-25 miR-210 miR-16	Pancreatic Juice	172 (54 PDAC, 118 NMC)	QIAzol Lysis Reagent (Qiagen, Hilden, Germany, #79306) and miRNeasy Mini kit (Qiagen, Hilden, Germany, #217004)	RT-qPCR	84.2	81.5	0.91	Diagnostic	3 (1 + 1 + 1)	Nesteruk et al.^62^
miR-21	Pancreatic Juice				NS	NS	NS			
miR-25					NS	NS	NS			
miR-210					NS	NS	NS			
miR-16					NS	NS	NS			
miR-155					NS	NS	NS			
miR-4516	Pancreatic Juice	26 (15 PDAC, 11 CP)	Ultracentrifugation	Custom miRNA array + RT-qPCR	80	80.8	NS	Diagnostic	5 (1 + 2 + 2)	Sakaue et al.^71^
miR-4674					81.8	73.3	NS			
Combined Panel (miR-4516 + miR-4674 + pancreatic juice cytology)					93.3	81.8	NS			
miR-3940-5p/miR-8069 ratio	Urine	92 (55 PDAC, 25 H, 12 CP)	Precipitation: ExoQuick TC (System Biosciences)	3D digital PCR	58.1	89.2	HCs versus PDAC AUC of 0.83	Diagnostic	1 (1 + 0 + 0)	Yoshizawa et al.^72^
45-miRNA panel (specific miRNAs not individually listed)	Urine	462 (153 PDAC, 309 H)	Polymer-based precipitation (commercial kit; specific brand not disclosed)	Small RNA-seq (Illumina platform)	93.9 (training), 77.8 (test)	91.7 (training), 95.7 (test)	0.972 (training), 0.963 (test)	Diagnostic	1 (1 + 0 + 0)	Baba et al.^73^
miR-20a	duodenal fluid	34 (27 PDAC, 7 H)	Ultracentrifugation	RT-qPCR	NS	NS	0.88	Diagnostic	4 (1 + 1 + 2)	Taniguchi et al.^74^

Abbreviations: AC, ampullary cancer; AVC, ampulla of Vater cancer; BCA, benign cyst adenoma; BPD, benign pancreatic disease; BPT, benign pancreatic tumour; CC, chronic cholecystitis; CCA, cholangiocarcinoma; CP, chronic pancreatitis; EV, extracellular vesicle; FBS, foetal bovine serum; GC, gastric cancer; H, healthy control; HCC, liver cancer; HNC, head and neck cancer; HSA, human serum albumin; IHC, immunohistochemistry; IPMN, intraductal papillary mucinous neoplasm; LC, lung cancer; MISEV, Minimal Information for Studies of Extracellular Vesicles; NGS, next-generation sequencing; NMC, non-malignant controls; NS, not stated; NSCLC, non-small cell lung cancer; OPT, other pancreatic tumour; PanNET, pancreatic neuroendocrine tumour; PC, pancreatic cancer; PDAC, pancreatic ductal adenocarcinoma; RT-qPCR, reverse transcription quantitative polymerase chain reaction; SEC, size exclusion chromatography; SERS, surface-enhanced Raman spectroscopy; UC, ultracentrifugation.

From the papers included in this review, the diagnostic potential of 3 miRNAs appeared to be investigated most commonly than: miR-21, miR-10b and miR-451a, with 13, 9 and 7 papers, respectively. The SN, SP and AUC for these 3 miRNAs from each of their respective papers are tabulated in Table 2. Scores representing each study’s adherence to a modified checklist reflecting the set criteria (Box 1) as well as a breakdown of these scores are also shown.

**Table 2.** Diagnostic Performance and EV Characterisation Scores of Studies Evaluating miR-21, miR-10b, and miR-451a as Biomarkers for Pancreatic Cancer.

miRNA biomarker	Total MISEV score breakdown	Sensitivity	Specificity	AUC	Biofluid	References
miR-21	7 (2 + 3 + 3)	80	90	0.869	Serum	Wu et al.^59^
	5 (1 + 2 + 2)	NS	NS	0.767	Blood	Yan et al. ^19^
	5 (1 + 2 + 2)	81	88	0.9	Pancreatic Juice	Nakamura et al.^69^
	3 (0 + 1 + 2)	NS	NS	0.771	Plasma + Pancreatic Cancer Tissue	Kim et al.^30^
	3 (0 + 1 + 2)	NS	NS	NS	Plasma + PDAC organoid deried EV	Zeöld et al.^34^
	3 (1 + 1 + 1)	100	100	1	Plasma	Lai et al.^35^
	3 (1 + 0 + 2)	NS	NS	0.93	Plasma	Feng et al.^41^
	3 (1 + 1 + 1)	NS	NS	0.002	Serum and Pancreatic Juice	Nesteruk et al.^62^
	2 (1 + 1 + 0)	NS	NS	0.733	Plasma	Xu et al.^42^
	2 (1 + 0 + 1)	72.7	72.7	NS	Plasma	Kawamura et al.^49^
	1 (1 + 0 + 0)	80.8	81	0.826	Serum	Goto et al.^53^
	1 (1 + 0 + 0)	NS	NS	NS	Plasma	Roy et al.^40^
	0 (0 + 0 + 0)	95.5	81.5	0.887	Serum	Que et al.^64^
miR-10b	5 (1 + 2 + 2)	90	100	0.98	Plasma	Yu et al.^37^
	4 (0 + 2 + 2)	NS	NS	NS	Plasma	Pang et al.^28^
	4 (1 + 1 + 2)	NS	NS	NS	Plasma	Joshi et al.^36^
	3 (0 + 1 + 2)	NS	NS	0.693	Plasma + Pancreatic Cancer Tissue	Kim et al.^30^
	3 (0 + 2 + 1)	NS	NS	0.6543	Plasma	Pu et al.^27^
	3 (1 + 1 + 1)	100	100	1	Plasma	Lai et al.^35^
	3 (1 + 0 + 2)	NS	NS	0.91	Serum	Madhavan et al.^63^
	3 (0 + 1 + 2)	NS	100	0.99	Plasma	Xiao et al.^47^
	2 (0 + 0 + 2)	NS	NS	0.996	Serum	Jiang et al.^55^
	5 (1 + 2 + 2)	NS	NS	0.793	Blood	Yan et al.^19^
miR-451a	5 (1 + 2 + 2)	NS	NS	0.9329	Serum	Verel-Yilmaz et al.^57^
	5 (2 + 2 + 1)	NS	NS	NS	Plasma	Vicentini et al.^49^
	3 (0 + 1 + 2)	80.1	86.67	0.896	Plasma	Chen et al.^52^
	2 (1 + 0 + 1)	72.7	77.3	NS	Plasma	Kawamura et al.^49^
	1 (0 + 0 + 1)	69.2	70.8	NS	Plasma	Takahasi et al.^31^
	1 (1 + 0 + 0)	65.6	85.7	0.789	Serum	Goto et al.^53^

Abbreviation: NS, not stated.

Across the 13 studies investigating **miR-21**, adherence to MISEV criteria was variable but relatively balanced compared to other two miRNAs (Table 2). Only 23.1% of studies demonstrated strong adherence, whilst the remainder were evenly split between moderate (38.5%) and weak (38.5%) adherence. Most studies with poor adherence failed to quantify EVs or lacked essential global or single-vesicle characterisation, often omitting protein markers or imaging. For example, studies by Kawamura et al.,^49^ Xu et al.^42^ and Que et al.^64^ either omitted EV quantification or both global and single-vesicle characterisation. In contrast, higher-scoring studies (eg, Yan et al.^19^ and Nakamura et al.^69^) implemented thorough EV characterisation but still lacked full details of biofluid preparation and quantification of EV yield, as recommended by MISEV guidelines. Despite showing a more balanced distribution of adherence scores, the overall variability limits the diagnostic robustness of EV miR-21 as a PDAC biomarker.

For **miR-10b**, adherence was skewed towards moderate quality, with 77.8% of studies (7 out of 9) scoring between 3 and 4 points (Table 2), with only 11.1% achieving strong adherence. Moderate-scoring studies often reported partial EV characterisation, such as the inclusion of single-vesicle analyses or selected protein markers but omitted full EV quantification. For example, Pang et al.^28^ failed to quantify EVs despite using Western blotting and TEM, while Joshi et al.^36^ employed 2 vesicle analysis techniques but only characterised 1 transmembrane protein. The predominance of moderate adherence suggests limited methodological robustness across the literature, contributing to the inconsistent diagnostic performance of EV miR-10b.

Studies assessing **miR-451a** showed the highest proportion of strong MISEV adherence of the 3 described miRNAs, with 42.9% of studies scoring 5 out of 8 (Table 2). However, an equal proportion (42.9%) of reports scored poorly (1-2 points). High-scoring studies implemented more complete EV characterisation, but low-scoring studies (eg, Takahashi et al.,^31^ Goto et al.^53^ and Kawamura et al.^49^) often failed to perform global EV characterisation or relied solely on single-vesicle approaches. This bimodal distribution, with clusters of both highly rigorous and poorly conducted studies, raises concerns regarding the reproducibility and generalisability of EV miR-451a-related findings.

Additional miRNAs, and in particular several miRNA panels, have demonstrated greater diagnostic potential than 3 biomarkers described above, as reflected in their higher AUC values. This is illustrated in Table 3.

**Table 3.** Diagnostic Performance and EV Characterisation Scores of Representative Studies Reporting Multi-miRNA Panels.

miRNA biomarker	Total MISEV score breakdown	Sensitivity	Specificity	AUC	References
miR-21 miR-25 miR-210 miR-16	3 (1 + 1 + 1)	84.2	81.5	0.91	Nesteruk et al.^62^
miR-103b, miR-23a-3p, miR-409-3p, miR-224-5p, miR-1299	1 (1 + 0 + 0)	88	95	0.95	Yang et al.^24^
miR-1246 miR-4644 miR-3976 miR-4306	1 (0 + 1 + 0)	NS	NS	0.958	Madhavan et al.^63^
miR-93-5p miR-339-3p miR-425-5p miR-425-3p	0 (0 + 0 + 0)	80	94.7	0.887	Makler et al.^43^
45-miRNA panel (specific miRNAs not individually listed)	1 (1 + 0 + 0)	93.9 (training), 77.8 (test)	91.7 (training), 95.7 (test)	0.972 (training), 0.963 (test)	Baba et al.^73^
miR-10b	3 (0 + 1 + 2)	NS	100	0.99	Xiao et al.^47^
miRNA-10b	2 (0 + 0 + 2)	NS	NS	0.99	Jiang et al.^55^

Abbreviation: NS, not stated.

Biomarker panels in general reported higher AUC values than individual miRNAs, with Nesteruk et al.,^62^ Yang et al.,^24^ Madhavan et al.,^63^ Makler et al.^43^ and Baba et al.^73^ all reporting AUCs exceeding 0.85. However, these panels consistently exhibited poor adherence to MISEV guidelines, with most scoring only 1 out of 8, primarily due to inadequate global and single-EV characterisation. Therefore, despite demonstrating promising diagnostic potential, the systematic omission of critical EV characterisation steps raises concerns about the reliability of reported diagnostic performance. Among individual miRNAs, **miR-10b** showed higher diagnostic performance (AUC = 0.99) than the panels in 2 independent studies (Xiao et al.^47^ and Jiang et al.^55^), but similarly suffered from weak methodological reporting (MISEV score of 3 and 2 respectively).

The risk of bias assessment using the QUADAS-2 tool^17^ revealed that most studies exhibited methodological concerns. A large proportion of studies were judged to have an unclear or high risk of bias in the domains of *patient selection* and *index test*, primarily due to incomplete reporting of inclusion criteria, sample handling and absence of blinding during sample analysis. 54/56 included papers lacked blinding and 37/56 studies did not incorporate independent validation cohorts, increasing the likelihood of overfitting and inflating diagnostic accuracy estimates. The reference standard domain was inconsistently addressed, with several studies failing to specify how PDAC diagnosis was confirmed. Flow and timing were generally adequately reported, although some studies lacked details regarding the timing of biomarker assessment relative to diagnosis. The breakdown of risk of bias assessments across all studies is summarised in Supplemental Figure 1. A new quality assessment checklist summarising recommended methodological standards for future EV-miRNA biomarker studies is provided in Supplemental Table 2.

## Discussion

This systematic review demonstrates that despite growing interest in EV-derived miRNAs as non-invasive biomarkers for PDAC, substantial methodological variability limits the interpretability and clinical utility of published findings. A prominent limitation lies in the inconsistent adherence to the MISEV guidelines, which recommend comprehensive characterisation of EV preparations. Majority of included studies failed to report global EV quantification using established metrics such as total protein or particle count, omitted key transmembrane (CD63, CD81, CD9) and cytosolic (TSG101, Alix) protein markers, and neglected single-EV characterisation altogether (Box 1). Such omissions raise the risk of non-EV contaminants like protein aggregates or lipoproteins confounding downstream analyses.^75-77^ This issue was particularly evident in studies investigating emerging miRNA candidates or miRNA panels, where reported diagnostic performance often exceeded AUC values of 0.90 despite minimal EV validation, which can result in misleading findings from contamination or technical errors.

Nonetheless, a subset of studies, particularly those evaluating miR-21and miR-451a did incorporate more rigorous methodological practices, including Western blotting for multiple EV markers and imaging via transmission electron microscopy. These studies tended to produce more reproducible results, highlighting the value of methodological robustness. For example, the most consistent findings for miR-21 came from studies scoring at least 5 out of 8 on the MISEV criteria, incorporating both global quantification and protein-based EV validation. However, the fact that miR-21 studies still exhibited wide variation in reported AUC values, ranging from <0.70 to >0.90 even among high-adherence groups, underscores the impact of other confounding factors, such as biofluid selection, normalisation strategies, and population heterogeneity. Most studies included patients with PDAC across all stages; with a small subset with restricted inclusion of early-stage (I–IIA/B). This variability may have influenced reported diagnostic performance and limited the ability to directly compare findings across studies. Similar heterogeneity was equally apparent in the diagnostic evaluation of miR-10b and miR-451a. For miR-10b, studies predominantly demonstrated moderate methodological adherence, often including some EV validation steps (eg, Western blotting or electron microscopy) without integrating quantification metrics or assessing contamination. These findings suggest that even promising biomarkers may yield misleading results if methodological standards are not uniformly applied.

Our findings collectively underscore an urgent need for standardisation across pre-analytical workflows, EV isolation methods, and miRNA quantification protocols. Harmonisation of these processes would enable more reliable cross-study comparisons and minimise the risk of misleading results arising from technical variability. To support this, journals and funding bodies should mandate compliance with minimum EV characterisation criteria, as outlined in the MISEV guidelines, to ensure methodological transparency and reproducibility.

Beyond EV validation, analytical variability introduced further uncertainty. RNA isolation methods, qPCR platforms and miRNA normalisation strategies were different and were rarely standardised or justified. Additionally, many studies failed to specify the total input volume of biofluid, a detail essential for contextualising miRNA concentration. Together, these factors introduce pre-analytical and analytical bias, which can distort comparisons across studies and compromise biomarker reliability.

Arguably the most critical study design limitation identified in this review was the widespread absence of external validation cohorts. Nearly 70% of included studies relied exclusively on discovery-phase data, with no attempt to assess the miRNAs in independent study populations. This introduces a significant risk of overfitting, whereby predictive performance appears inflated due to tailoring of results to the initial cohort. This limitation was particularly pronounced among studies reporting high AUC values, especially in those investigating miRNA panels, where model complexity increased but methodological transparency decreased. Thus, without proper separation of discovery and validation phases, these findings cannot be generalised.

The QUADAS-2 risk of bias assessment further substantiated these concerns. Nearly all studies lacked blinding during sample processing or analysis, introducing observer bias. Moreover, reference standards for confirming PDAC were often poorly defined or inconsistently applied, with some studies failing to specify whether diagnoses were histologically confirmed. Although the flow and timing of patient inclusion were generally well reported, the cumulative impact of these issues, especially when coupled with the absence of validation cohorts, undermines confidence in reported diagnostic accuracies.

The above-mentioned challenges reflect broader translational barriers that extend beyond EV-miRNA studies. As Peters et al. observed, fewer than 2% of candidate biomarkers in oncology ultimately reach clinical implementation.^78^ This failure is frequently attributed to the same issues identified here: inadequate methodological rigour, lack of reproducibility and insufficient multi-cohort validation. The Biomarker Toolkit^78^ has been proposed as a structured framework for addressing these gaps, offering a stepwise approach to evaluate biomarker readiness, from analytical validity to clinical utility. Incorporating such tools alongside standardised reporting frameworks like MISEV and QUADAS-2 could significantly improve both the design and interpretability of future studies.

## Conclusion

In summary, EV-derived miRNA biomarkers for PDAC offer significant diagnostic potential, particularly given their non-invasive nature, stability and biological relevance. However, current evidence base is undermined by methodological inconsistency, poor adherence to EV characterisation standards, and a pervasive lack of validation cohorts. These shortcomings have led to inflated diagnostic performance metrics and limited confidence in study findings. To advance the field, future research must prioritise methodological rigour through robust EV validation, use of independent multi-cohort designs and adoption of biomarker readiness frameworks. Only by addressing these limitations can EV miRNA biomarkers be credibly positioned for clinical translation for PDAC detection.

## Supplemental Material

sj-docx-1-bmi-10.1177_11772719251381960 – Supplemental material for Extracellular Vesicle-Derived miRNAs as Diagnostic Biomarkers for Pancreatic Ductal Adenocarcinoma: A Systematic Review of Methodological Rigour and Clinical Applicability Supplemental material, sj-docx-1-bmi-10.1177_11772719251381960 for Extracellular Vesicle-Derived miRNAs as Diagnostic Biomarkers for Pancreatic Ductal Adenocarcinoma: A Systematic Review of Methodological Rigour and Clinical Applicability by Ryhan Divyang Patel, Bhavik Patel and Tatjana Crnogorac-Jurcevic in Biomarker Insights
