# Supplementary material for: Extracellular Vesicle-Derived miRNAs as Diagnostic Biomarkers for Pancreatic Ductal Adenocarcinoma: A Systematic Review of Methodological Rigour and Clinical Applicability
Source: Biomark Insights. 2025 Oct 25;20:11772719251381960. doi: 10.1177/11772719251381960 (PMC12572613; doi:10.1177/11772719251381960)
Supplement: sj-docx-1-bmi-10.1177_11772719251381960 – Supplemental material for Extracellular Vesicle-Derived miRNAs as Diagnostic Biomarkers for Pancreatic Ductal Adenocarcinoma: A Systematic Review of Methodological Rigour and Clinical Applicability [file sj-docx-1-bmi-10.1177_11772719251381960.docx]

**Supplementary Table 1**- Search Strategy

| **MEDLINE (406)** | **Search** |
| --- | --- |
| 1 | exp Neoplasms/ |
| 2 | ("cancer*" or "carcinoma*" or "adenocarcinoma*" or "neoplasm*" or "tumour*" or "tumor*" or "malignanc*").af. |
| 3 | 1 or 2 |
| 4 | pancrea*.af. |
| 5 | exp Pancreas/ |
| 6 | 4 or 5 |
| 7 | 3 and 6 |
| 8 | exp MicroRNAs/ |
| 9 | ("microRNA*" or "miRNA*" or "short non-coding RNA*").af. |
| 10 | ("miR" or "microR").af. |
| 11 | 8 or 9 or 10 |
| 12 | exp Exosomes/ |
| 13 | exp Extracellular Vesicles/ |
| 14 | exp Microsomes/ |
| 15 | (exosome* or "extracellular vesicle*" or microsome* or microvesicle* or "membrane vesicle*" or "intracellular multivesicular endosome*").af. |
| 16 | 12 or 13 or 14 or 15 |
| 17 | 7 and 11 and 16 |

| **Cochrane Library (2)** | **Search** |
| --- | --- |
| 1 | MeSH descriptor: [Neoplasms] explode all trees |
| 2 | cancer OR cancers OR carcinoma OR carcinomas OR adenocarcinoma OR adenocarcinomas OR neoplasm OR neoplasms OR tumour OR tumours OR tumor OR tumors OR malignancy OR malignancies |
| 3 | #1 or #2 |
| 4 | MeSH descriptor: [Pancreas] explode all trees |
| 5 | pancreas or pancreatic |
| 6 | #4 or #5 |
| 7 | #3 and #6 |
| 8 | MeSH descriptor: [MicroRNAs] explode all trees |
| 9 | "microRNA" or "microRNAs" or "miRNA" or "miRNAs" or "short non-coding RNAs" or "short non-coding RNA" |
| 10 | "miR" or "microR" |
| 11 | #8 or #9 or #10 |
| 12 | MeSH descriptor: [Exosomes] explode all trees |
| 13 | MeSH descriptor: [Extracellular Vesicles] explode all trees |
| 14 | MeSH descriptor: [Microsomes] explode all trees |
| 15 | exosome or "extracellular vesicle" or microsome or microvesicle or "membrane vesicle" or "intracellular multivesicular endosome" or exosomes or "extracellular vesicles" or microsomes or microvesicles or "membrane vesicles" or "intracellular multivesicular endosomes" |
| 16 | #12 or #13 or #14 or #15 |
| 17 | #7 and #11 and #16 |

| **Embase (1187)** | **Search** |
| --- | --- |
| 1 | 'malignant neoplasm'/exp |
| 2 | 'cancer*' OR 'carcinoma*' OR 'adenocarcinoma*' OR 'neoplasm*' OR 'tumour*' OR 'tumor*' OR 'malignanc*' |
| 3 | #1 OR #2 |
| 4 | 'pancreas'/exp |
| 5 | 'pancrea*' |
| 6 | #4 OR #5 |
| 7 | #3 AND #6 |
| 8 | 'microrna'/exp |
| 9 | 'microrna*' OR 'mirna*' OR 'short non-coding rna*' |
| 10 | 'mir' OR 'micror' |
| 11 | #8 OR #9 OR #10 |
| 12 | 'exosome'/exp |
| 13 | 'microsome'/exp |
| 14 | 'exosome*' OR 'extracellular vesicle*' OR 'microsome*' OR 'microvesicle*' OR 'membrane vesicle*' OR 'intracellular multivesicular endosome*' |
| 15 | #12 OR #13 OR #14 |
| 16 | #7 AND #11 AND #15 |
| 17 | #7 AND #11 AND #15 AND [2000–2020]/py |
| 18 | #7 AND #11 AND #15 AND [2021–2022]/py |
| 19 | #7 AND #11 AND #15 AND [2023–2025]/py |

| **PubMed (398)** | **Search** |
| --- | --- |
| 1 | pancrea* |
| 2 | cancer* OR carcinoma* OR adenocarcinoma* OR neoplas* OR tumour* OR tumor* OR malignan* OR PC OR PDAC |
| 3 | mi*RNA* OR miR-* OR "short non-coding RNA" OR microR* OR "micro R*" |
| 4 | exosom* OR "extracellular vesicle*" OR EV* OR microsomes OR microvesicles OR membrane vesicles OR intracellular multivesicular endosomes |
| 5 | 1 AND 2 AND 3 AND 4 |

**Supplementary Figure 1**- QUADAS-2 Risk of Bias Assessment Summary


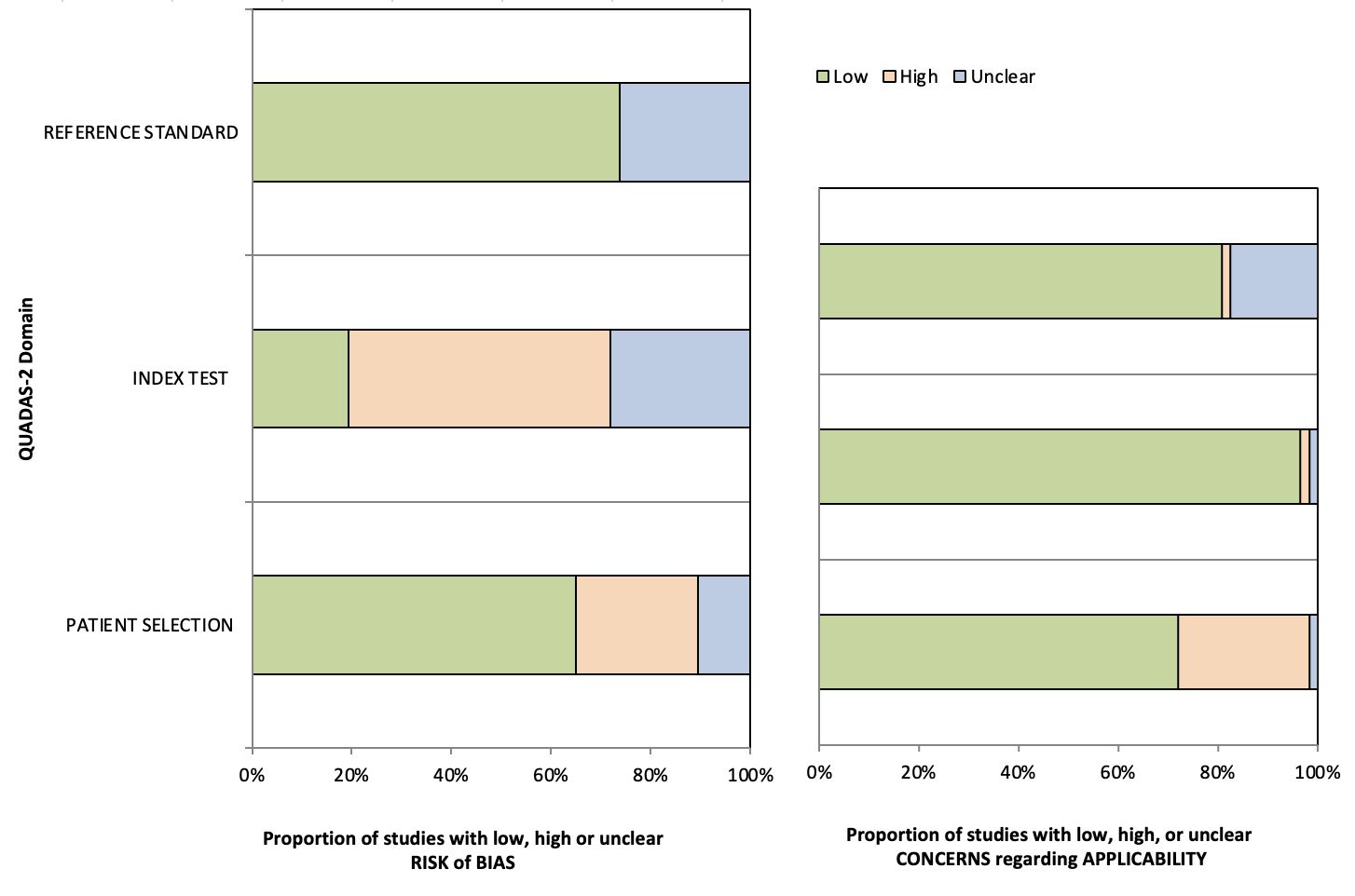
 Supplementary Figure 1: Graphical representation of QUADAS-2 Risk Of Bias assessment of the selected studies.

**Supplementary Table 2**- Quality Assessment Checklist for EV-based miRNA Diagnostic Studies

| **Domain** | **Requirement** | **Yes/No** |
| --- | --- | --- |
| **Study Design** | Prospective design with pre-specified criteria | ☐ |
|  | Blinding in sample processing and analysis | ☐ |
|  | External validation cohort included | ☐ |
| **Pre-analytical** | Biofluid volume recorded and reported | ☐ |
|  | Standardised storage and handling | ☐ |
| **EV Isolation & Quantification** | Isolation method validated and detailed | ☐ |
|  | Particle count via NTA/TRPS reported | ☐ |
|  | Protein amount via BCA reported | ☐ |
|  | Protein-to-particle ratio provided | ☐ |
|  | Contaminant assessment performed | ☐ |
| **EV Characterisation** | ≥1 transmembrane protein marker | ☐ |
|  | ≥1 cytosolic/membrane-binding protein | ☐ |
|  | Imaging of EVs performed | ☐ |
|  | Non-image single EV analysis performed | ☐ |
| **miRNA Analysis** | Extraction method described | ☐ |
|  | RNA quality assessed | ☐ |
|  | Normalisation strategy reported | ☐ |
| **Reporting & Validation** | SN, SP, AUC with 95% CI reported | ☐ |
|  | PRISMA & MISEV adherence | ☐ |
|  | Multi-cohort validation performed | ☐ |
